# The Effect of Naturally Occurring Chronic Kidney Disease on the Micro-Structural and Mechanical Properties of Bone

**DOI:** 10.1371/journal.pone.0110057

**Published:** 2014-10-15

**Authors:** Anna Shipov, Gilad Segev, Hagar Meltzer, Moran Milrad, Ori Brenner, Ayelet Atkins, Ron Shahar

**Affiliations:** 1 Koret School of Veterinary Medicine, Hebrew University of Jerusalem, Rehovot, Israel; 2 Department of Veterinary Resources, Weizmann Institute, Rehovot, Israel; Université Jean Monnet, France

## Abstract

Chronic kidney disease (CKD) is a growing public health concern worldwide, and is associated with marked increase of bone fragility. Previous studies assessing the effect of CKD on bone quality were based on biopsies from human patients or on laboratory animal models. Such studies provide information of limited relevance due to the small size of the samples (biopsies) or the non-physiologic CKD syndrome studied (rodent models with artificially induced CKD). Furthermore, the type, architecture, structure and biology of the bone of rodents are remarkably different from human bones; therefore similar clinicopathologic circumstances may affect their bones differently. We describe the effects of naturally occurring CKD with features resembling human CKD on the skeleton of cats, whose bone biology, structure and composition are remarkably similar to those of humans. We show that CKD causes significant increase of resorption cavity density compared with healthy controls, as well as significantly lower cortical mineral density, cortical cross-sectional area and cortical cross-sectional thickness. Young's modulus, yield stress, and ultimate stress of the cortical bone material were all significantly decreased in the skeleton of CKD cats. Cancellous bone was also affected, having significantly lower trabecular thickness and bone volume over total volume in CKD cats compared with controls. This study shows that naturally occurring CKD has deleterious effects on bone quality and strength. Since many similarities exist between human and feline CKD patients, including the clinicopathologic features of the syndrome and bone microarchitecture and biology, these results contribute to better understanding of bone abnormalities associated with CKD.

## Introduction

Chronic kidney disease (CKD) is a growing public health concern worldwide, with increasing incidence in all age groups. The prevalence of moderate to severe CKD in the general population is reported to be as high as 8.5% [1], [2]. The disease is irreversible and progressive in nature, and as it progresses, metabolic derangements worsen. This is particularly true in the ageing population, where CKD has become a major cause of morbidity and mortality.

CKD-associated bone diseases include several different types of bone pathologies, such as adynamic bone disease and osteomalacia which are characterized by low bone turnover, osteitis fibrosa cystica which is characterized by high bone turnover (due to secondary hyperparathyroidism) and mixed uremic osteodystrophy which is characterized by either high or low turnover and abnormal mineralization [3].

One of the inevitable metabolic consequences of CKD is secondary renal hyperparathyroidism (SRH) [4]. The pathophysiology of SRH is complex and involves phosphorus retention leading to hyperphosphatemia, ionized hypocalcemia, decreased circulating 1,25-dihydroxyvitamin D (calcitriol) concentration and increased concentrations of parathyroid hormone (PTH) and fibroblast growth factor 23 [FGF23, [5]]. FGF23, which has been shown to have a pivotal role in mineral homeostasis, is produced mainly by osteocytes and osteoblasts [6]. Serum levels of FGF23 increase already in the early stages of CKD, when patients are still normo-phosphatemic and have normal PTH levels [7]–[9]. When PTH levels increase, they promote bone resorption, and persistently high PTH concentrations, as documented in CKD patients, eventually lead to, osteopenia, and increased risk of pathological fractures [10].

It is widely recognized that bone fragility increases markedly in patients with CKD, and that fracture risk increases with progression of the disease [11]–[13]. The risk of pathological fractures has been reported to increase by 9% with each 200-pg/mL increase in PTH concentration, and by 72% with PTH concentrations above 900 pg/mL (reference range, 150–300 pg/mL), [10], Furthermore, the United States Renal Data System identified a 4-fold greater risk of hip fractures in human dialysis patients as compared to the general population [11].

The precise structural and compositional changes in the skeleton that occur in CKD patients, are not entirely clear. Data regarding the nature of these changes is crucial to the understanding of the skeletal consequences of CKD, since they determine the quality of bone material and the quantitative deterioration of material's mechanical properties. Most previous studies were based on data collected from human patients or from rodent models. For obvious reasons, studies conducted on human patients are subjected to severe inherent limitations, primarily the need to rely on non-invasive or minimally invasive (biopsy) methods. Biopsies, while a valuable diagnostic tool, by their nature provide data relevant to a very small region in a bone, and therefore can provide only limited information. Noninvasive methods used in human patient studies, such as determination of bone mass (or apparent mineral density) by dual x-ray absorptiometry (DEXA) provide imprecise information because of technical limitations, as described eloquently by Parfitt [14]. The main limitations of DEXA include its reliance on a 2-D proxy of mineral density (g/cm^2^) measurement rather than true 3-D density, and the inability to obtain separate data for cortical and trabecular bone. Peripheral quantitative computed tomography (pQCT) provides true 3-D information and is therefore a valuable tool, however its resolution is in the mm range.

On the other hand, use of model animals (almost exclusively mice and rats), while allowing the use of a wide array of testing methods, is hindered by the fact that in most studies, CKD is induced by non-physiologic means, mostly partial nephrectomy [15]. This obviously does not mimic with precision the disease in human patients, and may affect the skeleton in ways which are subtly (or even substantially) different from those caused by the natural course of the disease in humans. Moreover, the structure and architecture of rat and mouse cortical bone differs greatly from that of human cortical bone, as shown recently by Shipov *et al* and Bach-Gansmo *et al* [16], [17]. Therefore, the ability to directly extend the observed effects of artificially-induced CKD in the rat skeleton to the effects of naturally-occurring CKD in the human skeleton is limited.

The course, pathology and pathophysiology, diagnosis and treatment of feline CKD mirror those of the human disease very closely, and the disease is very prevalent in the feline population [18]. Another major advantage of studying the effects of CKD in cats is that the bones of mature cats are structurally and compositionally very similar to those of humans, both consisting mostly of remodeled secondary osteons [19].

Here we present a detailed study of the skeletal changes, both structural and mechanical, in cats with naturally occurring CKD. In this study we compare the femora and vertebra of cats diagnosed with CKD and those of age-matched cats with normally functioning kidneys.

## Materials and Methods

### 2.1 Animals and data collection

The study was prospective, based on the patient population of the Veterinary Teaching Hospital of the Hebrew University of Jerusalem, and was approved by the institutional animal care and use committee. Cats considered for the study either died or were euthanized at their owners' request after medical management had failed. Euthanasia was performed using 200 mg/kg pentobarbital (CTS chemical industries LTD, Israel) administered intravenously. Cats were enrolled only after their owners had signed an informed consent form and donated the body to science. The study group consisted of 13 cats diagnosed with Stage III or IV CKD, based on the classification scheme of the International Renal Interest Society guidelines [20], for at least 6 months prior to death or euthanasia. These criteria included documentation of persistent azotemia (3 occasions, at least 2 weeks interval, serum creatinine concentration >2.8 mg/dL), urine specific gravity <1.020 and ultrasonographic changes consistent with CKD. CKD was additionally confirmed in all cats by histopathological examination showing moderate to severe interstitial nephritis accompanied by moderate to severe fibrosis.

The control group included 13 healthy cats without any clinicopathologic signs of CKD (e.g., normal creatinine, concentrated urine) that died or were euthanized in the Veterinary Medical Teaching Hospital due to reasons unrelated to diseases of the urinary system. Cats with concurrent metabolic diseases that could potentially affect the skeleton were excluded, as were cats that were treated for more than 2 weeks during the 6 months prior to their death with medications that could alter bone metabolism (e.g., vitamin D derivatives, corticosteroids).

### 2.2 Sample collection and preparation

Blood and serum samples from all cats were collected *antemortem* for complete blood count and biochemical analysis. Sera were stored at −80°C for determination of PTH and vitamin D levels.

Tissue sample collection was performed within 12 hours of death. The right femora and lumbar vertebrae were carefully removed and cleaned of all soft tissue, wrapped in saline-soaked gauze, placed in a sealed plastic bag and stored at −20°C until testing. Kidney samples were harvested and stored in 10% formalin for histologic evaluation.

### 2.3 Light microscopy

Thin transverse slices (400 microns thick) of the mid-diaphyseal region of all right femora were cut by a water-cooled slow-speed diamond saw (Buehler Isomet low Speed saw, USA). The slices were then polished by increasingly fine grit (Buehlet Minimet Polisher, USA), from 320 grit to 1 µm diamond paste. Transverse cross-sections of all cortical samples were viewed by reflective light microscopy (Olympus BX-51) and their detailed architecture characterized by analysis of images captured by a dedicated high-resolution camera (Olympus DP 71, 12 MegaPixels).

**Figure 1:**
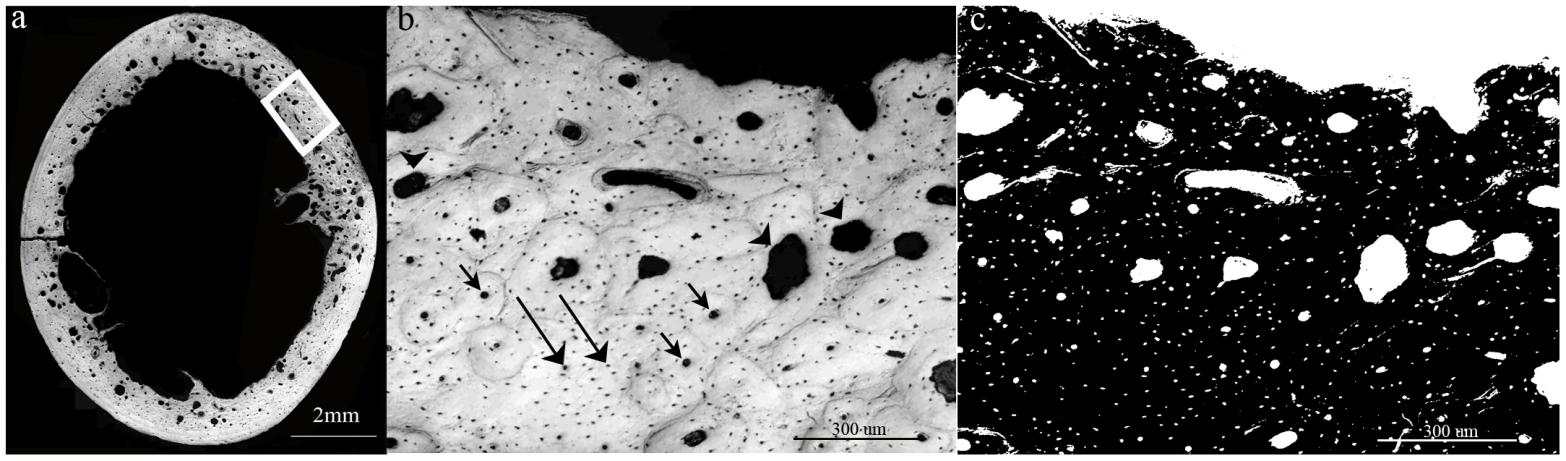
Bone cavity analysis. (a) Light microscopy image of a typical transverse cross section of the bone created by stitching together of many individual images. Classification of voids was performed on based on their size. (b) An individual image from the cross sectional image (marked by a white rectangle in image a). (c) Each image was first binarized, separating it into ‘bone’ (white) and ‘void’ (black) (right image). Cavities within the range of 9–50 µm^2^ were considered to be lacunae (long arrows), cavities within the range of 151–2000 µm^2^ were considered to be Haversian canal (short arrows) and cavities larger than 2000 µm^2^ were considered resorptive lesions (arrowheads).

Quantitative analysis of the transverse cross-sectional images, particularly quantification of voids and their classification, was performed with a public domain image processing software (ImageJ, NIH, v. 1.44p). Several microstructural parameters were measured, such as the number, size and density of the osteocytic lacunae, Haversian canals and resorption cavities within each cross section [21]. Specifically, each cross-sectional image was first binarized by selection of an appropriate threshold, separating it into ‘bone’ (white) and ‘void’ (black) entities [22], (Fig. 1 a, b). Next, the ImagJ ‘analyze particles’ command was applied to each cross-section to identify all individual voids within it. This command analyzes each void by its size and reports the results in a tabular form (see Figure 1) [23]. Based on sizes of osteocytic lacuna and Haversian canals reported in the literature [24]–[27] voids with an area in the range of 9–150 µm^2^ were considered to be osteocytic lacunae, voids with an area in the range of 151–2000 µm^2^ were considered to be Haversian canals, while voids larger than 2000 µm^2^ were considered resorption cavities which are in the process of remodeling [21]. Voids smaller than 9 µm^2^ were considered to be artifacts. Images were also visually examined by two of the authors (AS and HM) and the results of thresholding and void categorization were manually corrected if indicated. Overall porosity was calculated as the ratio of total void area (i.e. resorption cavities, lacunae and blood vessels) to total bone area.

### 2.4 Mechanical testing

Mechanical properties of cortical bone were evaluated using four-point bending tests performed on bone beams prepared from the cranial aspect of the mid-diaphyseal cortical region of the right femora. Beam sizes were 20 mm×1.5 mm×1 mm (long dimension along the bone axis).

Mechanical testing was performed with the samples immersed in saline, using a custom-built micromechanical-testing device as previously described [28]. All samples were thawed immediately before testing for one hour at room temperature. The beams were placed within a saline-containing testing chamber that had a stationary anvil attached to its wall [28]. This anvil consisted of two supports which were 15 mm apart. A movable double-pronged loading anvil was attached to a load-cell (model 31, Honeywell Sensotec, Colombus, OH, USA), which was in turn attached to a high-precision linear motor (PI GmbH, Karlsruhe, Germany). The loading anvil had a span of 5 mm between its two prongs, which were centered between the two supports of the stationary anvil.

The upper prongs were brought into contact with the tested beams at a predetermined preload (2N), the chamber was filled with physiologic saline solution at room temperature until the samples were fully immersed, and bending tests were conducted under displacement control at a rate of 500 µm/180 seconds up to failure. Force-displacement data were collected by custom-written software (LabView, National Instruments, Texas, USA) at 50 Hz. Load and displacement values were converted to stress and strain, respectively, based on beam theory [29]. The stress-strain curves were used to estimate Young's modulus of the beam material, as well as yield and failure stresses and strains. It should be noted that care was taken to minimize shear deformation at the supports by maintaining a ratio of distance between supports/beam depth of 15∶1 [30], [31]. Yield point was determined for each beam as the point at which a line parallel with the linear portion of the stress-strain curve and offset by 0.03% strain intersected with this curve [32].

### 2.5 Microstructural characterization by Micro-CT

All cortical beams, the right femur and the 6^th^ and 7^th^ lumbar vertebrae were scanned by microCT (Skyscan 1174 compact micro-CT scanner, Belgium), with the beams scanned prior to mechanical testing. Analyses were performed on the entire beam, the mid-diaphyseal femoral cortex (cortical bone analysis), and in the distal femoral metaphyses and vertebral bodies (cancellous bone analysis).

The X-ray source was set at 50 kVp and 800 µA. A total of 450 projections were acquired over an angular range of 180°. The samples were scanned with an isotropic voxel size of 11.1 µm for the cortical bone beams and 19.6 µm for the femoral cortex and cancellous bone of both the femora and vertebrae. Integration time for all scans was 4500 ms, and a 0.25 mm aluminum filter was used. Scans were reconstructed and analyzed using commercial software (NRecon Skyscan software, version 1.6.1.2 and CT analyser Skyscan software, version 1.9.3.2, respectively). Cortical bone mineral density (BMD) of the beams was determined based on calibration with 2 phantoms of known mineral density (0.25 g/cm^3^ and 0.75 g/cm^3^) supplied by SkyScan, which were scanned under exactly the same condition as were the bone specimens.

### 2.6 Statistical analysis

The distribution of continuous parameters (normal vs. non-normal) was assessed using the Shapiro-Wilk's test. Normally and non-normally distributed continuous parameters were compared between the study and the control group using Student's t-test and Mann-Whitney U test, respectively. Gender proportion between the study group and the control groups was compared using the Fischer Exact test. Correlations between continuous parameters (e.g., biomechanical parameters and PTH concentration) were performed using the Pearson or the Spearman Rank correlation test, according to data distribution. For all tests *P*<0.05 was considered statistically significant. All calculations were performed using a statistical software (SPSS 17.0 for Windows, SPSS Inc; Chicago, IL, USA).

## Results

### 3.1 Animals

The study population included 26 cats, of which 13 were diagnosed with CKD and 13 were healthy controls. There were eight males and five females in the study group and six males and seven females in the control group, with no gender proportion differences between the study groups. Mean body weight was significantly lower in cats with CKD compared with healthy controls (2.8±0.6 kg vs. 3.7±0.9 kg, respectively; *P* = 0.01). There was no statistically significant difference in mean age between the study and control groups (10.5±5.6 years compared to 9.7±3.9 years, respectively; *P* = 0.7).

### 3.2 Clinical pathology

Median serum creatinine concentration within the CKD group was 8.2 mg/dL (range 3.5–16.0 mg/dL) compared with a median of 0.9 mg/dL (range 0.6–1.2 mg/dL) [reference interval (RI), 0.5–1.6 mg/dL] of the control group. Three cats in the study group (23%) were classified as Stage III CKD, and the rest (77%) were classified as Stage IV CKD. Median phosphorous concentration in the study group was 8.8 mg/dL (range 5.3–21.7 mg/dL; RI, 3.0–6.2 mg/dL). Median concentration of ionized calcium in the study group was 0.80 mmol/L (range 0.65–1.01 mmol/L; RI, 0.9–1.4 mmol/L). PTH concentration, available for five cats of the study group, had a median concentration of 15.70 pmol/L (range, 0.9–32.9 pmol/L; RI, 0.4–2.5 pmol/L). Vitamin D concentration was below normal in five out of the seven cats in which it was measured (median 63 nmol/L, range 35–143 nmol/L; RI 65–170 nmol/L).

### 3.3 Cortical bone architecture

**Figure 2:**
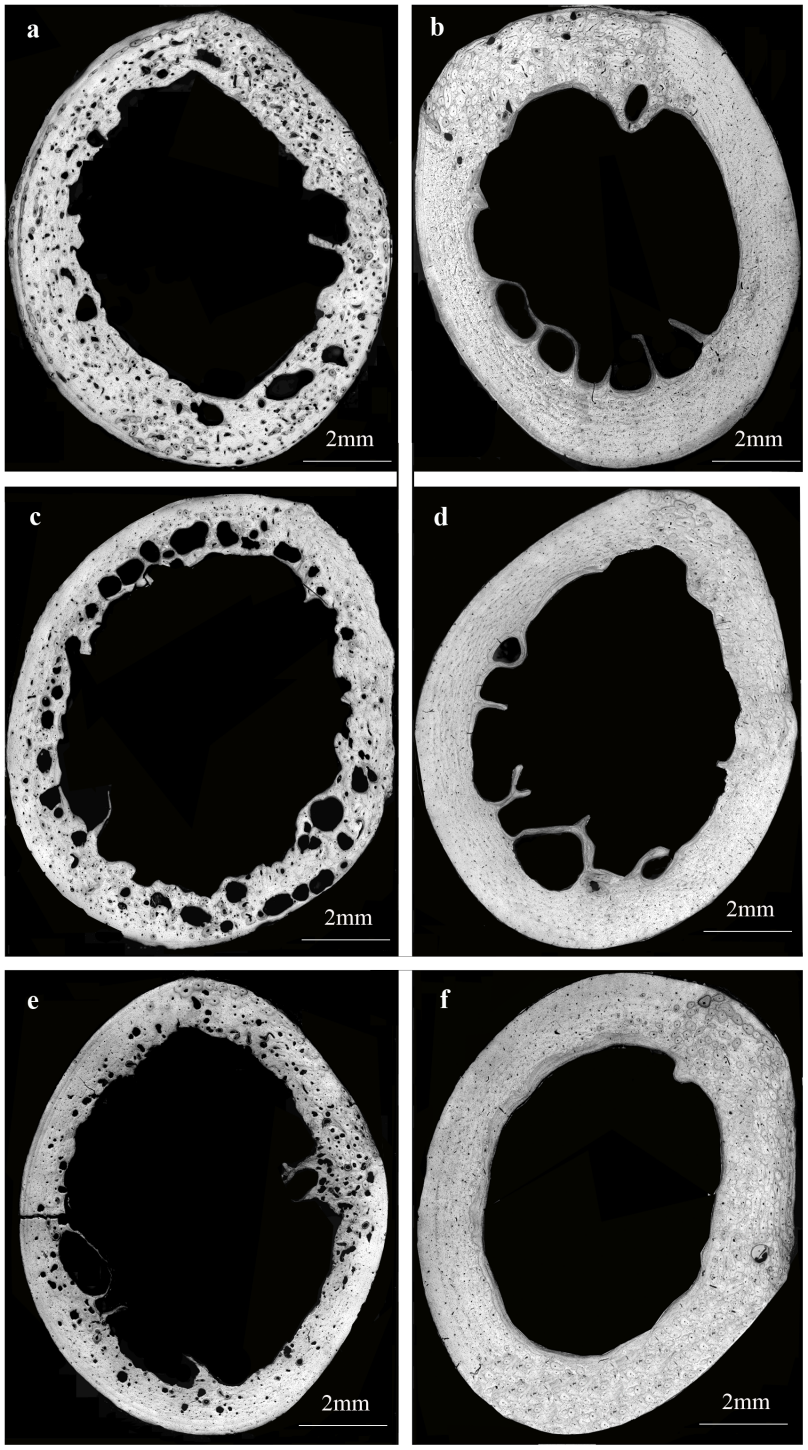
Light microscopy images of three transverse cross-sections of the femoral mid-diaphysis of (a, c, e) cats with CKD and (b, d, f) healthy cats. Note dramatic increase in unfilled resorption cavities in CKD cats compared to the healthy cats.

**Figure 3:**
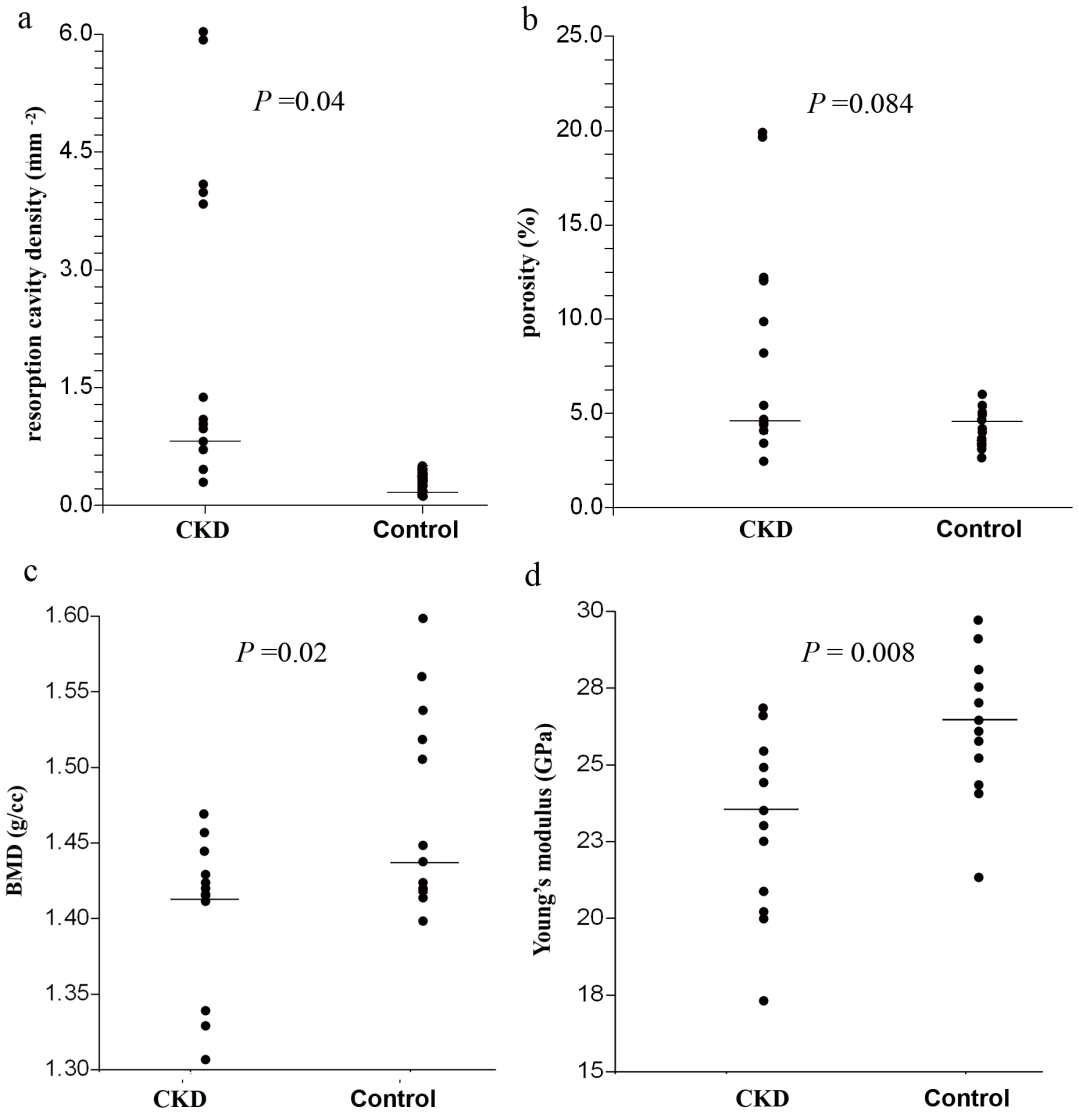
Dot plots depicting the data of CKD and control cats; the horizontal line represents the median. (a) resorption cavity density (b) porosity, (c) BMD and (d) Young's modulus.

The results of the architectural analysis are presented in Table 1 (microscopy) and Table 2 (microCT). CKD-affected cats had significantly higher density of resorption cavities compared to healthy controls (Table 1, Figures 2, 3a). Other structural parameters were not significantly different between the groups. Porosity tended to be higher in the CKD group, however the difference between groups did not reach statistical significance (*P* = 0.084, Figure 3b).

**Table 1:** Morphometric characteristics of cortical bone of the distal femur in CKD and healthy controls by light microscopy.

Light microscopy morphometry	CKD (mean ±SD)	Controls (mean ±SD)	*P* value
*Oseteocytic lacunae*			
Size [µm^2^]	33.9±3.1	33.1±3.9	0.60
Density [mm^−2^]	510±55	524±106	0.70
*Haversian canals*			
Size [µm^2^]	481±115	411±48	0.10
Density [mm^−2^]	22.3±4.3	23.3±4.6	0.58
*Resorption cavities*			
Size [µm^2^]	10,342±11,888	12,406±25,258	0.78
Density [mm^−2^]	2.2±2.4	0.4±0.1	**0.04**

**Table 2:** Morphometry of cortical bone of the mid-diaphyseal femur in CKD and healthy controls.

Microtomography	CKD (mean ±SD)	Controls (mean ±SD)	*P* value
Cortical cross sectional area (mm^2^)	28.5±3.6	32.2±5.5	**0.04**
Cross-sectional thickness (mm)	1.1±0.2	1.2±0.2	**0.03**
Mean polar area of inertia (µm^4^)	432±95	520 ±154	0.08

Micro-CT analysis of cortical bone of the femoral diaphysis showed significantly lower cortical cross-sectional area and cross-sectional thickness in CKD cats (Table 2). Mean polar area moment of inertia tended to be lower in the CKD group, but the differences did not reach statistical significance (*P* = 0.083) (Table 2).

### 3.4 Cortical mineral density

Cortical mineral density of CKD cats was lower by 4.8% compared to controls, (*P = *0.02, as shown in Figure 3c).

### 3.5 Mechanical properties of cortical bone

Table 3 and Figure 3 present a comparison of several mechanical properties of cortical bone material between the CKD and control groups. Bones from the CKD group were shown to have inferior mechanical properties compared to the control group, in particular lower stiffness (Young's modulus), lower yield stress, and lower ultimate stress (Table 3, Figure 3).

**Table:** **Table 3.** Mechanical properties of the cortical bone of CKD and control groups.

Parameter	CKD (mean ±SD)	Controls (mean ±SD)	*P* value
**Young's modulus (GPa)**	**23.5±2.9**	**27.1±2.8**	**0.01**
**Yield stress (MPa)**	**151±18**	**166±14**	**0.04**
Yield strain (millistrain)	5.6±0.8	5.6±0.4	.95
**Ultimate stress (MPa)**	**185±16**	**205±15**	**0.01**
Ultimate strain (millistrain)	8.6±1.0	8.1±0.4	0.37

Correlation could not be demonstrated between PTH levels and any of the mechanical properties of the cortical bone.

### 3.6 Architecture of trabecular bone

**Figure 4:**
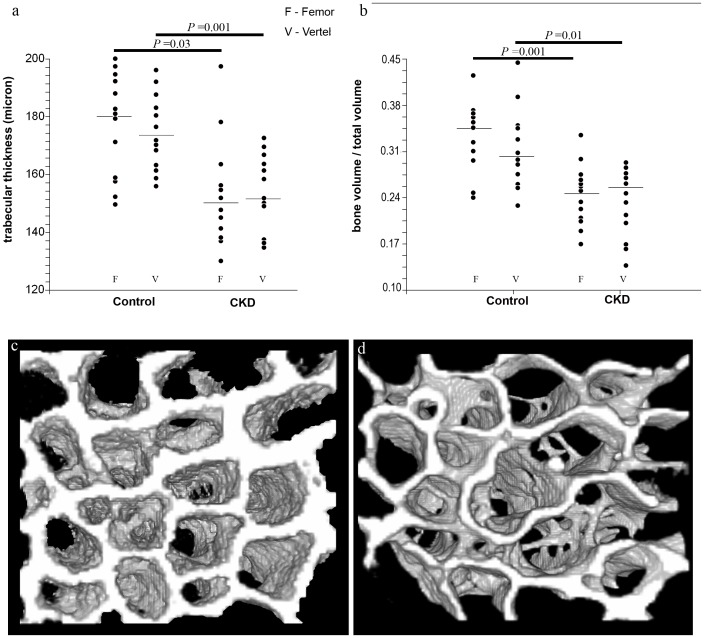
Trabecular bone analysis: Trabecular thickness (a) and bone volume/total volume (BV/TV, b) in the cancellous bone of CKD and control cats for both the femora (F) and the vertebrae (V). Data are presented as dot plots. The horizontal line represents the median. Data from the femur and vertebra are similar within the study groups; however, there is a significant difference for both parameters between the study and the control groups. Micro-CT scans of cancellous bone of a control (c) and CKD (d) cat.

Analysis of cancellous bone in the 6^th^ and 7^th^ lumbar vertebrae and in the distal femur revealed significantly lower trabecular thickness and bone volume over total volume (BV/TV) in bones belonging to the CKD group, compared with control cats (Figure 4).

## Discussion

This study demonstrates that advanced CKD in cats results in deterioration of bone quality, in particular a dramatic increase of resorption cavities and decreased bone mineral density. These results provide insight into skeletal changes occurring in human CKD due to the similarity between cats and humans in terms of the pathophysiology of the syndrome and the type of bone architecture.

To the best of our knowledge, this is the first study to measure the detailed mechanical and structural effects of CKD on the skeleton of an animal model with *naturally occurring* CKD. Feline and human CKD have very similar clinicopathologic features and progression, therefore cats are superior models compared to rodents in which pharmaceutical and surgical interventions are usually employed to induce kidney disease [33]–[36]. Furthermore, the cat skeleton shows great similarities to human bone, in particular because it remodels continuously throughout life and therefore consists mostly of secondary osteons. Rodent bones on the other hand are remarkably different from those of human bones in terms of type, architecture, structure and biology. Most dramatically, rodent cortical bone does not remodel [16], [17]. Therefore similar clinicopathologic circumstances, such as those occurring due to CKD, may affect rodent bones differently from human (or cat) bones.

We found that naturally occurring CKD results in several alterations to the architecture and morphology of the bones of the skeleton. Cortical thickness was found to be decreased by approximately 17% in CKD cats compared with controls. This change compromises the mechanical performance of long bones, by reducing their flexural stiffness and is likely to lead to increased fracture risk. Previous studies conducted in human patients with CKD showed similar tendencies, however with much smaller changes. For instance, a recent study documented 4.2% increase in porosity, 2.9% decrease in cortical area and 2.8% decrease in cortical thickness, indicating progressive loss of cortical bone [37]. These results, like those of other human studies, were based on DEXA and high resolution peripheral quantitative CT. These methods have been shown to be limited in precision in terms of bone volume quantification due to inability to separate cortical from cancellous bone (DEXA), and low-resolution volumetric measurements, compared to microCT and whole bone sampling [38].

Overall cortical porosity in cats with advanced CKD tended to be somewhat higher compared with controls (Figure 3b), but this difference did not reach statistical significance, most likely due to small group size and biological variation. However, the density (number per unit area) of resorption cavities in CKD patients is greatly increased (5-fold, 2.22/mm^2^ vs. 0.41/mm^2^, *P* = 0.04). Such a difference is expected to affect mechanical behavior of long bones radically, and is likely to play an important role in the increased fragility of CKD patients. Previous studies in a rat model demonstrated that persistently elevated PTH concentrations result in high bone turnover, exhibited as elevated numbers and size of osteoclasts, increased osteoblastic activity and enhanced bone resorption [33]. Consequently, these studies found extensive endocortical, intracortical and periosteal resorption, resulting in a dramatic increase in cortical porosity (9.75% compared to 0%). It should be noted however that these results were observed in rat bone, which normally does not remodel, as oppose to cat (and human) bone.

Bone mineral density is another major determinant of bone quality, and the current clinical standard for prediction of fracture risk in osteoporotic patients [39]. Therefore, determining the influence of CKD on BMD was a major objective of this study. The cortical mineral density of cats with CKD was significantly lower compared to controls. It should be noted that despite appearing small, this decrease (4.8%) is clinically significant, as even a small decrease in BMD substantially decreases the stiffness of the bone and increases fracture risk [40], since the relationship between them is exponential [41]. A decrease in BMD was also reported in various studies in humans CKD patients, with a wide range of values [1.3% to 17.5%; [37], [42], [43]]. However, these studies were based on areal BMD (g/cm^2^, using DEXA), histomorphometry or pQCT, while the current study measured volumetric BMD at high resolution and precision using microCT [38], [44].

Reliable measurement of material properties requires precise and accurate mechanical testing. Such testing is difficult to achieve in rodents due to the small size of their bones, which are often tested by bending tests of whole bones, using the 3-point bending technique. Results are dependent upon the geometry of the bones and mechanical properties of the material, often leading to underestimation of Young's modulus [45], [46]. The size of cat bones allowed us to prepare cortical bone beams, enabling accurate and reliable assessment of the material properties using four-point bending testing. Furthermore, four-point bending tests of beams allowed us also to measure other material properties, like yield and strength, and to document significant decrease in yield stress and failure stress in the cortical bone of the CKD cats compared to controls.

This study demonstrates that the cortical bone material of CKD cats is less stiff and more prone to micro-damage which occurs at lower loads, and will result in increased bone fragility. In particular, Young's modulus of the cortical bone, which reflects the stiffness of the material, was shown here to be significantly lower in the CKD group (23.5 GPa vs 27.1 GPa, respectively, *P* = 0.008). The lower Young's modulus in CKD cats compared with controls is most likely due to a combination of higher porosity and lower BMD.

Cancellous bone is also significantly affected by CKD, as reflected by decreased trabecular thickness and lower bone volume (BV/TV). Furthermore, the effect on cancellous bone was multi-site, shown to occur both in the vertebral bodies as well as in the long bones. Both changes (trabecular thickness and BV/TV) negatively affect bone quality and were shown to be associated with increased risk for fracture [47].

Since this study was based on cats with naturally occurring kidney disease, some variability existed among them in terms of the severity and the chronicity of the disease. Furthermore, the number of cats included in this study was relatively small, though comparable to numbers typically seen in published rodent model studies. It should also be noted that cats with advanced CKD often exhibit decreased appetite and thus might fail to consume enough food to meet their caloric requirements. Such nutritional deficiencies, as in human patients, might contribute to their decreased bone quality. Additionally, all of the cats in this study had a single etiology (interstitial nephritis, which is the etiology in more than 70% of cases with CKD in cats [18]), whereas human patients with CKD have multiple etiologies (e.g., diabetic nephropathy, transplant patients, etc.). Nevertheless, slowly progressive natural disease represents the human syndrome and its consequences, including renal osteopathy, much better than artificially-induced rodent models.

In conclusion, the current study demonstrates the deleterious effects of CKD on remodeled (secondary osteonal) bone quality and strength, including increased bone resorption, decreased BMD, and inferior mechanical and structural properties. Due to these changes, the bones of CKD patients become more fragile. Since many similarities have been demonstrated between human and feline CKD patients, in terms of the clinic-pathologic features of the syndrome, as well as bone-associated effects, cats are an extremely suitable and relevant animal model for studying the development of bone abnormalities in humans suffering from CKD.
